# Establishment and optimization of the two‐step induction system for generating primordial germ cell‐like cells from chicken embryonic stem cells

**DOI:** 10.1002/2211-5463.70116

**Published:** 2025-09-10

**Authors:** Zeyu Li, XianShuai Xu, GuangZheng Liu, XiaoQian Lv, JiuZhou Song, HongYan Sun, YingJie Niu, QiSheng Zuo, Wei Han, BiChun Li, Kai Jin

**Affiliations:** ^1^ Joint International Research Laboratory of Agriculture and Agri‐Product Safety of Ministry of Education of China Yangzhou University China; ^2^ Key Laboratory of Animal Breeding Reproduction and Molecular Design for Jiangsu Province, College of Animal Science and Technology Yangzhou University China; ^3^ Institutes of Agricultural Science and Technology Development Yangzhou University China; ^4^ Department of Animal & Avian Sciences University of Maryland College Park MD USA; ^5^ College of Bioscience and Biotechnology Yangzhou University China; ^6^ Poultry Institute Jiangsu Institute of Poultry Sciences/Chinese Academy of Agricultural Sciences Yangzhou China; ^7^ College of Biotechnology Jiangsu University of Science and Technology Zhenjiang China

**Keywords:** chicken, embryonic stem cells, optimization, primordial germ cell‐like cells, two‐step induction system

## Abstract

Primordial germ cells (PGCs) are the progenitor cells of sperm and eggs. Xenotransplantation of chicken PGCs can achieve germline transmission. However, there are still challenges in obtaining many PGCs from endangered birds *in vitro*. In this study, at first, by incorporating 2i factors, the embryonic stem cells (ESCs) culture conditions were optimized, successfully yielding and validating pluripotent ESCs clones. Then, during induction ESCs, bFGF, activin A, and 1% KSR were added to Epiblast‐like cells (EpiLCs). Quantitative real‐time polymerase chain reaction (qRT‐PCR) showed Pax6, Eomes, and Vimentin expression patterns similar to primary epiblast, indicating successful EpiLCs induction. During EpiLCs to Primordial germ cell‐like cells (PGCLCs) transformation, we evaluated BMP4, BMP8b, EGF, LIF, and SCF combinations' impact on induction efficiency. Flow cytometry, qRT‐PCR, and immunofluorescence showed high expression of Cvh, C‐kit, Dazl, CVH, and DAZL in PGCLCs, suggesting successful EpiLCs differentiation. Induced PGCLCs injected into 2.5‐day chick embryos migrated to gonads by day 7–7.5, demonstrating migration and colonization. This study optimized a two‐step protocol for *in vitro* differentiation of chicken ESCs into PGCLCs. This research's results not only provide a reference for obtaining many PGCLCs *in vitro* but also open up a new approach for the development and application of genetic resource preservation technology in domestic chickens.

## Graphical abstract

This study optimizes a two‐step induction system to generate PGCLCs from chicken ESCs. The induced cells express germline markers and migrate to gonads *in ovo*, offering a novel approach for avian genetic resource conservation.

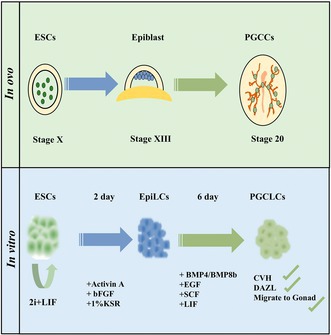

**Abbreviations**
AKP: alkaline phosphatase
BMP4: bone morphogenetic protein 4
BMP8b: bone morphogenetic protein 8b
cESCs: chicken embryonic stem cells
CVH: chicken Vasa homolog
DAPI: 4′,6‐diamidino‐2‐phenylindole
DAZL: deleted in azoospermia like
EGF: epidermal growth factor
EpiLCs: epiblast‐like cells
ERK: extracellular signal‐regulated kinase
ESCs: embryonic stem cells
FBS: fetal bovine serum
FGFR: fibroblast growth factor receptor
KIT: cluster of differentiation 117
KSR: knockout serum replacement
LIF: leukemia inhibitory factor
MEK: mitogen‐activated protein kinase kinase
PBS: phosphate‐buffered saline
PBST: phosphate‐buffered saline with tween‐20
PGCLCs: primordial germ cell‐like cells
PGCs: primordial germ cells
qRT‐PCR: quantitative real‐time polymerase chain reaction
SCF: stem cell factor
SSEA‐1: stage‐specific embryonic antigen‐1
TGFβ: transforming growth factor β

Primordial germ cells (PGCs) have become an effective tool for studying poultry transgenesis [1]. They also hold broad application prospects for preserving rare and endangered avian germplasm resources [2]. However, it is difficult to obtain a large number of PGCs *in vitro*, whether in mammals or avian species, thus making the study of their conversion from other types of cells into primordial germ cells important. Lu *et al*. explored the process of converting chicken embryonic fibroblasts into Primordial germ cell‐like cells (PGCLCs); however, due to the complex genetic background of fibroblasts, it increased the uncertainty and difficulty in the induction and differentiation process, leading to a complicated induction procedure and lower efficiency [3]. Embryonic stem cells (ESCs) are highly undifferentiated cells with the ability to self‐renew and possess the potential for multilineage differentiation. Studies have demonstrated that ESCs can differentiate into PGCLCs under appropriate conditions.

In 2011, Hayashi *et al*. [4] induced mouse adherent ESCs into Epiblast‐like cells (EpiLCs) using Activin A and bFGF and, subsequently, induced EpiLCs into PGCLCs using BMP4, BMP8b, EGF, SCF, and LIF. In 2012, the same research group reported the two‐step induction of PGCLCs from female ESCs, which were able to further develop into oocytes and produce offspring [5]. Based on Hayashi's work, Zhou *et al*. [6] adopted the same two‐step method to induce PGCLCs and obtained offspring through intracytoplasmic sperm injection (ICSI). Li [7] successfully transformed mouse adherent ESCs into spermatogonial stem cell‐like cells (SSCLCs) and sperm‐like cells. After transplantation into infertile male mice, these SSCLCs successfully produced healthy offspring. Yukiko [8] derived PGCLCs using the same two‐step method, which they later expanded for epigenetic reprogramming. The PGCLCs in the reconstructed testes could differentiate into robustly proliferating germ stem cell‐like cells (GSCLCs) with appropriate androgenic epigenomes. Oikawa [9] also utilized a dual‐stage process to induce functional PGCLCs from rat iPSCs. After transplantation into the seminiferous tubules of rats with reproductive defects, these PGCLCs produced functional sperm and fertile offspring. Although some progress has been made in inducing chicken ESCs(cESC) to differentiate into PGCLCs *in vitro*, such as the work by Gong and others where cESCs were first induced to differentiate into embryoid bodies (EBs) and then further into PGCLCs, the induction efficiency remains low due to the undirected differentiation of EBs, and the culture system has not been fully optimized [10].

In this study, based on the two‐step method in mammals, we successfully established and optimized a chicken PGCLCs induction model using key factors affecting chicken PGCs development and inhibitors of signaling pathways. This model not only efficiently generates PGCLCs with PGCs characteristics, but also endows these cells with allogeneic migration ability. In summary, our results pave the way for the preservation of avian genetic resources using cESCs and PGCLCs.

## Materials and methods

### Ethics statement

All of the procedures involving the care and use of animals conformed to the U.S. National Institute of Health guidelines (NIH Pub. No. 85‐23, revised 1996) and were approved by the Laboratory Animal Management and Experimental Animal Ethics Committee of Yangzhou University (No. 202202146). Fertilized breeder eggs (Rugao yellow chicken) were obtained from the Poultry Research Institute (Chinese Academy of Agricultural Sciences, Yangzhou, China) under an environment of 37 °C with 70% humidity.

### Cell isolation and culture

The Poultry Research Institute of the Jiangsu Institute of Poultry Sciences provided freshly fertilized eggs from *Gallus gallus* domesticus. We used blastoderm cells from the embryonic region of the EGK X stage fertilized eggs to isolate ESCs [11]. The specific steps are as follows: First, wash the fresh fertilized eggs successively with 1% benzalkonium bromide and 75% alcohol and then place them in a clean bench. Crack the eggshell at the equatorial plane, discard the upper half of the eggshell, remove the egg white, and keep the yolk. Locate the blastodisc and move it to the center of the yolk. Cut the vitelline membrane containing the blastodisc into a quadrilateral shape, then remove it and put it into a Petri dish filled with PBS. Shake the Petri dish to separate the blastodisc from the vitelline membrane. Suck the blastodisc into another Petri dish filled with PBS, wash it several times, and then collect it in a centrifuge tube. Disperse the blastodisc into a single‐cell suspension, filter it into a new tube, centrifuge it at 200 ***g*** for 6 min, and discard the supernatant. Resuspend the cells with the culture medium, and inoculate them into a Petri dish for cultivation.

The isolated cells were cultured in an optimized medium named ESC medium, which contains: KO‐DMEM (Gibco, Carlsbad, CA, USA, 12660012), 10% Knockout™ SR (Gibco, 10828028), 0.1 mmol·L^−1^ β‐mercaptoethanol (Sigma‐Aldrich, St. Louis, MO, USA, 21985023), 1 mmol·L^−1^ sodium pyruvate (Sigma, 11360070), 2 mmol·L^−1^ L‐GlutaMax (Gibco, 35050061), 1% nonessential amino acids (Gibco, 11140050), 1000 IU·mL^−1^ LIF (Sigma, L5158), 10 ng·mL^−1^ bFGF (Gibco, 13256‐029), 5 ng·mL^−1^ SCF (Sigma, SRP3151), 2% chicken serum (Gibco, 11140050), and 2i (MEK inhibitor PD0325901 and TGFβ inhibitor SB431542, MCE). This medium was prepared by adding the 2i factor on the basis of previous research [12]. The separation time and site of the chicken epiblast refer to the research conducted by Nayernia [13, 14]. The culture medium for epiblast cells contains KO‐DMEM (Gibco, 12660012), 20% Knockout™ SR (Gibco, 12660012), 2% chicken serum (Gibco, 11140050), 1 mmol·L^−1^ sodium pyruvate (Gibco, 11360070), 2 mmol·L^−1^ L‐GlutaMax (Gibco, 35050061), 1% nonessential amino acids (Gibco, 11140050), 0.1 mmol·L^−1^ β‐mercaptoethanol (Gibco, 21985023), and 20 ng·mL^−1^ LIF (Sigma, L5158). PGCs were isolated from the genital ridge of chicken embryos and incubated for 5.5 days. The cultivation protocol is based on the study conducted by Liu [15]. ESCs, epiblast, and PGCs from chickens were kept in a 37 °C, 5% CO_2_ environment. The methods for getting ESCs and PGCs have been explained before [16].

### The identification of ESCs

For alkaline phosphatase (AKP) staining (Solarbio, Beijing, China, BC2145), cells were washed with PBS (Solarbio, P1020), fixed in AKP fixative for 3 min, and washed again with PBS. Cells were then incubated with AKP staining solution for 15–20 min in the dark followed with PBS wash. The nucleus was counterstained with nuclear fast red/methyl green for 3–5 min and followed with PBS wash. Visualization of cellular and tissue structure was performed under an inverted microscope.

For indirect immunofluorescence, after removing ESC medium, cells were trypsinized, centrifuged, and washed with PBS. Then, cells were fixed with 4% paraformaldehyde (Sigma, 158127), permeabilized with TritonX‐100 (Solarbio, IT9100), and blocked with 10% FBS (Cytiva, Marlborough, MA, USA, SH30406.02). After the incubation with Anti‐SSEA‐1 (Abcam, Cambridge, UK, ab16285) at 4 °C overnight and then an incubation with Goat anti‐mouse IgG H&L Alexa Fluor^®^ 488 (Abcam, ab150113) at 37 °C in dark, cells were stained with DAPI (Solarbio, C0065) and observed under fluorescence microscope. For qRT‐PCR, the total RNA from ESCs was extracted using Trizol (Invitrogen, Carlsbad, CA, USA, 15596018CN). A cDNA library was constructed with the reverse transcription kit from Nanjing Novozymes Biotech Co, Ltd. (Nanjing, China). The sequences of the primers used to perform the quantitative real‐time PCR (qPCR) are listed in Table 1.

**Table 1:** Primer sequences used for quantitative real‐time PCR (qRT‐PCR) in ESCs identification.

Gene	No.	Primer sequences (5′‐3′)
*NANOG*	NM_001146142.2	F:GTATGCAACCAGCTCACC R:TAGTAGTGTCCGCACCTAAC
*SOX2*	NM_205188.3	F:CATATGTAAGACAAAGGGG R:AAGGTCCAGAATTTCTAATAA
*OCT4*	NM_001252452.1	F:TGAGAACCTTCAGGAGATAT R:TGATTGGCGATGTGAGTGA
*ACTB*	NM_205518.2	F:CAGCCATCTTTCTTGGGTAT R:CTGTGATCTCCTTCTGCATCC

### Differentiating ESCs into EpiLCs

We chose to use optimally cultured P3ESCs for the induction process. Culture ESCs in the EpiLCs induction medium, whose components include: Knockout DMEM (Gibco, 12660012), 1% Knockout™ SR (Gibco, 10828028), 25 ng·mL^−1^ Activin A (PeproTeh, Rocky Hill, NJ, USA, GMP120‐14F), 10 ng·mL^−1^ bFGF (Gibco, 13256‐029), 1 mmol·L^−1^ sodium pyruvate (Sigma, 11360070), 2 mmol·L^−1^ L‐GlutaMax (Gibco, 35050061), 1% nonessential amino acids (Gibco, 11140050), 0.1 mmol·L^−1^ β‐mercaptoethanol (Gibco, 21985023). The culture medium was changed daily.

### The identification of EpiLCs

After replacing the chicken EpiLCs induction medium, we collected cells on days 0, 1, 2, and 3 [10] of the induction processes (on the day of the first collection of EpiLCs, the first, second, and third days of induction culture) for qRT‐PCR analysis. Total RNA was extracted from the induced cells and reverse‐transcribed into cDNA. qRT‐qPCR was performed to detect the expression of genes related to the three germ layers, pluripotency, and lineage specificity during the induction process. The sequences of primers used for qRT‐PCR are shown in Table 2. *PAX6*, *VIM*, and *EOMES* were used to detect the three germ layers; *NANOG* and *OCT4* were used as pluripotency genes; *CVH, KIT*, and *DAZL* were used as lineage‐specific genes; and the chicken *ACTB* gene was used as an internal reference. SSEA‐1 indirect immunofluorescence was performed on the formed EpiLCs after 2 days of induction.

**Table 2:** Primer sequences used for quantitative real‐time PCR (qRT‐PCR) in EpiLCs identification.

Gene	NO.	Primer sequences (5′‐3′)
*PAX6*	NM_001397296.1	F:TGCCGCCCATGCCCAGCT R:ATGGGCTGGCTATTCATG
*VIM*	NM_001048076.3	F:GGACCTGCTGAATGTAAAGA R:AGGTTGGAATAGGCATGTTA
*EOMES*	XM_426003.7	F:GCCGCTCTGGCTCAAGTT R:CAGCACCACCTCCACGAA
*NANOG*	NM_001146142.2	F:GTATGCAACCAGCTCACC R:TAGTAGTGTCCGCACCTAAC
*OCT4*	NM_001252452.1	F:TGAGAACCTTCAGGAGATAT R:TGATTGGCGATGTGAGTGA
*CVH*	NM_204708.3	F:TTCTTGTGGCAACTTCGG R:AACTTCCTGCTGGGCTTC
*KIT*	NM_204361.1	F:GCATCCAGCAATGGTGAC R:AAGTTGCGTTGGGTCTAT
*DAZL*	NM_204218.2	F:TGTCTTGAAGGCCTCGTTTG R: CATATCCTTGGCAGGTTGTTGA
*ACTB*	NM_205518.2	F:CAGCCATCTTTCTTGGGTAT R:CTGTGATCTCCTTCTGCATCC

### Differentiating EpiLCs into PGCLCs

The EpiLCs induced for about 2 days were selected for further induction. The original medium was discarded and replaced with a PGCLCs induction medium. They were maintained in culture with medium replenishment every 2 days. The composition of the different induction groups media for PGCLCs was formulated by adding different inductive factors to the essential PGCLCs medium, with specific ingredients prepared according to Table 3.

**Table 3:** Formulation of induction medium for chicken PGCLCs.

Medium	Formula
Basic PGCLCs medium	KO‐DMEM (Gibco, 12660012) + 15%Knockout™ SR (Gibco, 10828028) + 1%double antibiotics+1 mmol·L^−1^ sodium pyruvate (Sigma, 11360070) + 2 mmol·L^−1^ L‐GlutaMax (Gibco, 35050061) + 1% nonessential amino acids (Gibco, 11140050) + 0.1 mmol·L^−1^ β‐mercaptoethanol (Gibco, 21985023).
Condition 1	40 ng·mL^−1^ BMP4 (MCE, HY‐124697) + 40 ng·mL^−1^ BMP8b (MCE, HY‐RS01544) + 50 ng·mL^−1^ EGF (Gibco, PHG0311)
Condition 2	40 ng·mL^−1^ BMP4 (MCE, HY‐124697) + 40 ng·mL^−1^ BMP8b (MCE, HY‐RS01544) + 50 ng·mL^−1^ EGF (Gibco, HG0311) + 1000 IU·mL^−1^ LIF (Sigma, L5158) + 10 ng·mL^−1^ SCF (Sigma, SRP3151)
Condition 3	40 ng·mL^−1^ BMP4 (MCE, HY‐124697) + 50 ng·mL^−1^ EGF (Gibco, PHG0311) + 1000 IU·mL^−1^ LIF (Sigma, L5158) + 10 ng·mL^−1^ SCF (Sigma, SRP3151)
Condition 4	40 ng·mL^−1^ BMP4 (MCE, HY‐124697) + 40 ng·mL^−1^ BMP8b (MCE, HY‐RS01544) + 1000 IU·mL^−1^ LIF (Sigma, L5158) + 10 ng·mL^−1^ SCF (Sigma, SRP3151)

### The identification of PGCLCs

qRT‐PCR: After replacing the chicken PGCLCs induction medium, cells from four induction groups were collected on days 2, 4, 6, and 8. We extracted total RNA from the induced cells of different groups and then reverse‐transcribed it into cDNA. qRT‐PCR was performed to detect the expression of lineage‐specific genes during the induction process. The primer design is shown in Table 2, with *CVH, KIT*, and *DAZL* as the detection primers for lineage‐specific genes and the chicken *ACTB* gene as the internal reference. Flow cytometry (BD Biosciences, Franklin Lakes, NJ, USA): Cells from four induction groups on day 6 were collected, digested with trypsin, centrifuged, washed with PBS, and transferred to 1.5 mL centrifuge tubes. The cells were then subjected to Triton X‐100 permeabilization treatment and blocked with 10% FBS‐PBS to ensure complete cell processing. Afterwards, the cells were incubated with the CVH primary antibody overnight at 4 °C and, subsequently, with the corresponding secondary antibody at 37 °C. Following this, the cells were resuspended in PBS, filtered in flow tubes, and detected using a flow cytometer with a negative control (unstained) as a reference. On the 6th day, we collected cells from the four induction groups to perform indirect immunofluorescence detection on the induced PGCLCs. The proteins detected by the immunofluorescence antibodies were CVH and DAZL for 2 h and then at 4 °C overnight. After washing three times with PBST, the cells were incubated with the corresponding secondary antibodies at 37 °C for 2 h without light, and then washed three times with PBST, followed by incubation with DAPI for 10 min without light, and then washed again. Fluorescence was observed and photographed under the fluorescence microscope (Leica Microsystems CMS GmbH Ernst‐Leitz‐Str, Shanghai, China).

### The identification of long‐term cultured PGCLCs

Using the FACS [15] system for the long‐term culture of PGCs and PGCLCs. Perform indirect immunofluorescence assays on P8 PGCLCs, 40‐day PGCs, and 180‐day PGCs to detect the expression of specific antibodies CVH (Abcam, ab13840), DAZL (Abcam, ab34139), and SSEA‐1 (Abcam, ab16285).

### Detection of PGCs and PGCLCs migration

Chicken PGCs and long‐term cultured PGCLCs were washed with serum‐free culture medium and centrifuged, then resuspended in diluent C. Subsequently, the cells were stained with PKH26, the reaction was terminated with serum, and multiple washes removed excess dye. Finally, they were resuspended in a complete medium and adjusted to an appropriate density for injection. After sterilization, freshly fertilized eggs are placed into the incubator with the large end facing up. At 2.5 days of embryonic development, the eggs were retrieved, and the blunt end was swabbed with alcohol and opened. The chicken embryo's blood vessels were then identified under a microscope, and a cell suspension of 5000 cells·μL^−1^ density was injected into the blood vessels using a microinjection needle. After dipping in a solution of penicillin and streptomycin, the opening was sealed. The eggs continue to incubate until the 7th day, when they are retrieved, and the embryos are extracted. The embryos were then cleaned in a petri dish containing PBS and antibiotics, and the abdominal cavity was dissected to isolate the genital ridge carefully. Under a stereo fluorescence microscope (Leica Microsystems CMS GmbH Ernst‐Leitz‐Str.), the fluorescence was observed, photographed, and recorded.

### Statistical analysis

All experiments were repeated three times and analyzed using graphpad prism 8 software for one‐way ANOVA (*P < 0.05, indicating a significant difference; **P < 0.01, indicating a highly significant difference) and graphing.

## Results

### The cultivation and identification of chicken ESCs

**Fig. 1:**
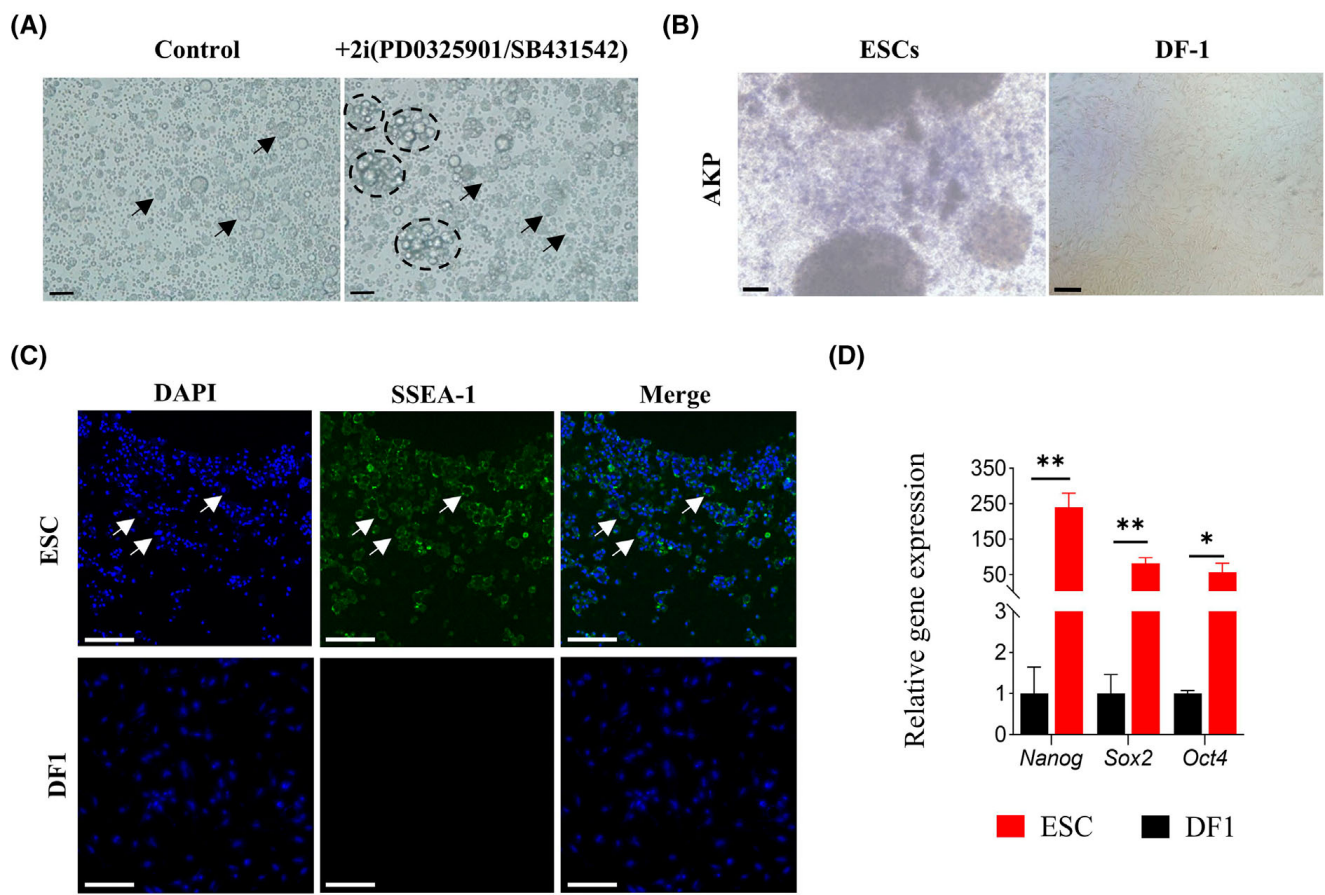
The cultivation and identification of chicken ESCs. (A) Morphological observation of cells cultured for 24 h under the original culture system (scale bar = 20 μm). (B) AKP results of ESCs and negative control DF1 cells (scale bar = 20 μm). (C) Immunofluorescence staining results of SSEA‐1 on ESCs and negative control DF1 cells (scale bar = 50 μm). (D) Quantitative detection of the expression of pluripotency genes, Data are presented as mean ± SD (*n* = 3 *P < 0.05, indicating significant difference; **P < 0.01, indicating extremely significant difference).

The results of cytomorphological observation during the culture process of optimized media showed that the clones of chicken ESCs exhibited a more typical shape, a greater number, and were more prone to aggregate into colonies (Fig. 1A). The cultured ESCs were positive for AKP and SSEA‐1, while no specific fluorescence staining was observed in the negative control DF1 cells (Fig. 1B,C). The qRT‐PCR results indicated that the expression levels of pluripotency marker genes *Nanog*, *Sox2*, and *Oct4* in ESCs were significantly different from those in DF1 [17] (*P* = 0.000483, *P* = 0.000794, and *P* = 0.019995, Fig. 1D). These results indicate that the *in vitro* cultured chicken embryonic stem cell clones, from the perspective of cell morphology, exhibit a typical and easily aggregated undifferentiated morphology. The positive results of AKP and SSEA‐1 in the cell surface antigen detection indicate that they have the characteristics of early‐stage embryonic cells and have not undergone specific differentiation. At the gene expression level, the high expression of pluripotency marker genes such as *Nanog*, *Sox2*, and *Oct4* implies that the cells can maintain an undifferentiated state and have the potential for multidirectional differentiation. In summary, the embryonic stem cell clones remain in an undifferentiated state and still possess the ability to express totipotency (i.e., the potential to differentiate into various cell types), which can be used for subsequent experiments.

### The differentiation and identification of chicken ESCs into EpiLCs

**Fig. 2:**
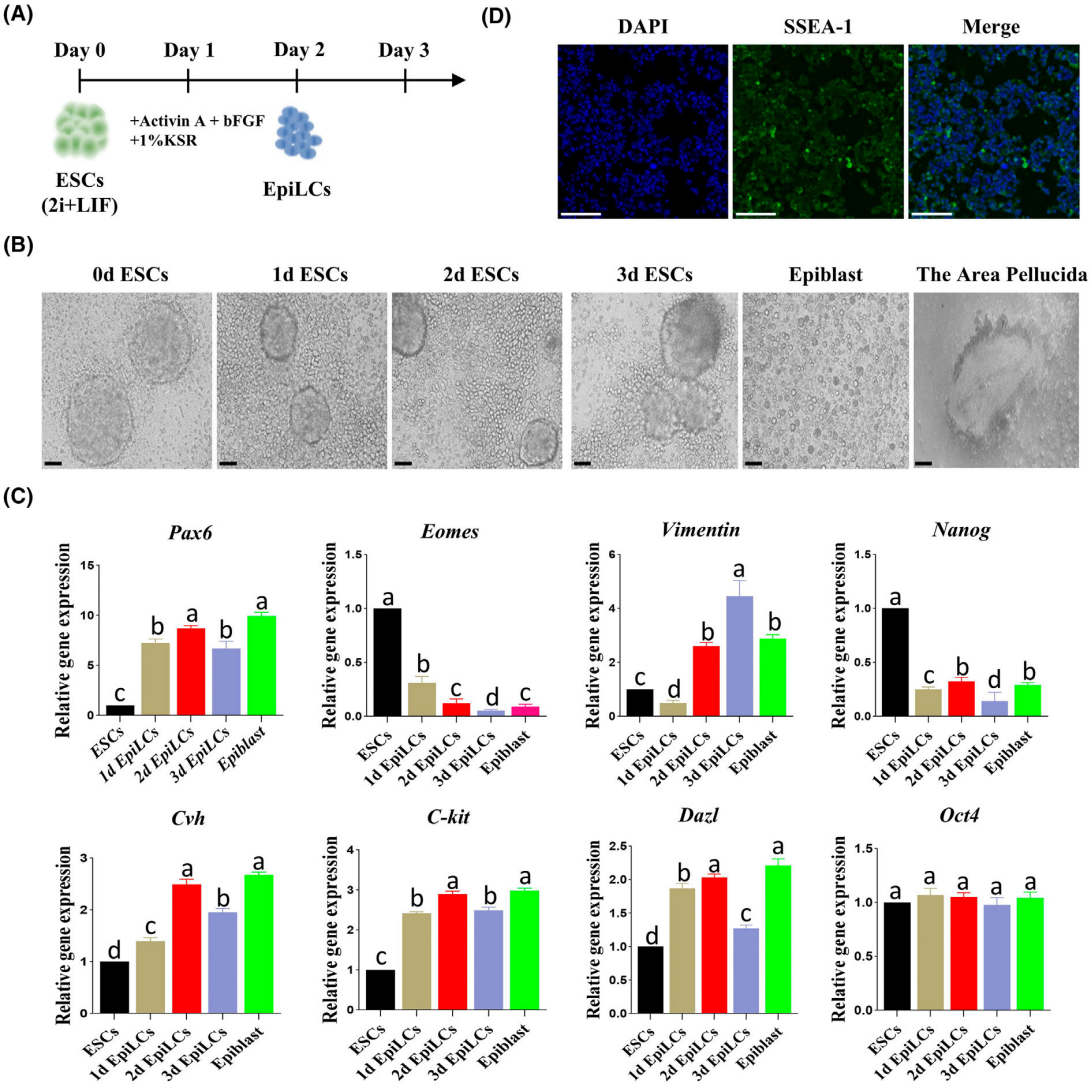
The differentiation and identification of ESCs into EpiLCs: (A) Induction protocol for chicken EpiLCs. (B) Respectively, the cell morphology images on day 0, 1, 2, and 3 during the ESC induction process, the image of primary chicken Epiblast cells isolated *in vitro*, and the transparent area of the embryo disk where the primary chicken Epiblast was isolated (scale bar = 20 μm). (C) Quantitative detection of the expression of genes related to EpiLCs (*n* = 3, Lowercase letters a, b, c indicate significant statistical differences between groups, analyzed by one‐way ANOVA with Tukey's multiple comparison test *P* < 0.05). (D) Immunofluorescence staining result of SSEA‐1 on EpiLCs (scale bar = 50 μm).

To establish a two‐step PGCs induction model, this study utilized a system of Activin A, bFGF, and 1% KSR to induce the *in vitro* differentiation of cESCs into EpiLCs (Fig. 2A). Cellular morphological results showed that the cells rapidly grew and developed into monolayer adherent, interconnected colonies within the first 2 days of induction. On the third day, the ability of the cell colonies to adhere decreased, and the cell number was significantly reduced. Therefore, it is speculated that the optimal time for the formation of chicken EpiLCs is on the second day of induction (Fig. 2B). To further explore the optimal time for the formation of EpiLCs, we detected the expression of the outer ectodermal‐specific gene *Pax6* and the mesoderm‐related genes *Eomes* and *Vimentin*, pluripotency genes (*Nanog* and *Oct4*), and lineage‐specific related genes (*Cvh*, *Kit* and *Dazl*) during the induction process. The qRT‐PCR results showed that the expression patterns of the formed EpiLCs in terms of three germ layer marker genes, pluripotency marker genes, and lineage‐specific marker genes were like those of primary Epiblast, and the EpiLCs induced for 2 days showed the highest similarity to primary Epiblast (Fig. 2C). Immunofluorescence detection was performed on the induced EpiLCs for 2 days to examine the expression of surface antigen SSEA‐1. Under fluorescence microscopy, it was observed that the EpiLCs formed after 2 days of induction could still be labeled by the specific protein SSEA‐1, with the cell membrane appearing green and the cell nucleus appearing blue after DAPI staining (Fig. 2D). This indicates that the EpiLCs formed after 2 days of induction still possess totipotency. It is proven that under the induction of the medium, ESCs can be induced to differentiate into EpiLCs, and the EpiLCs induced on 2 days show the best effect; thus, they were chosen for subsequent experiments.

### The differentiation of chicken EpiLCs into PGCLCs

**Fig. 3:**
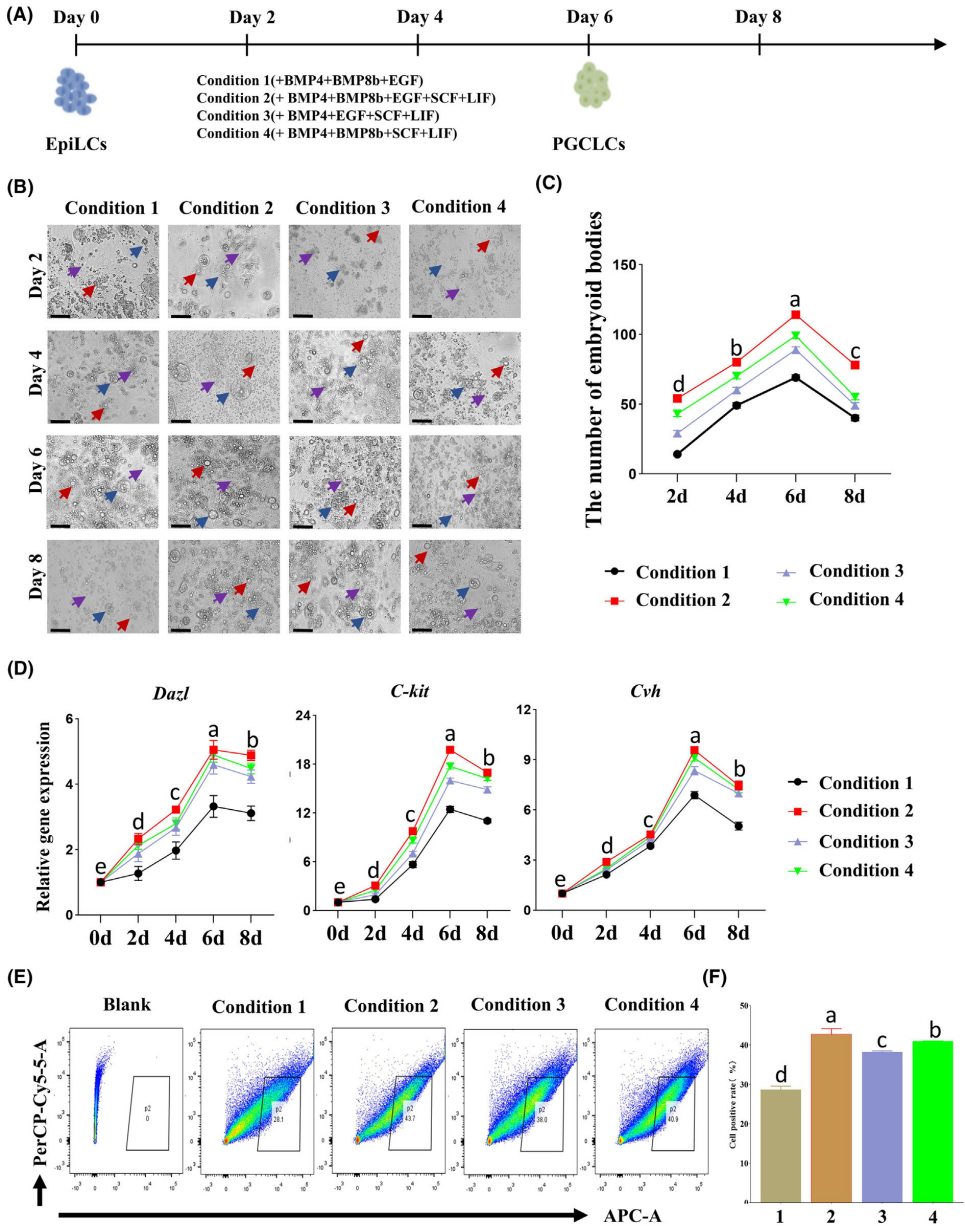
The differentiation of EpiLCs into PGCLCs: (A) Induction protocol for chicken PGCLCs. (B) Morphological observation of cells (scale bar = 20 μm). Among them, the purple arrow represents EpiLCs, the blue arrow represents EBs, and the red arrow represents PGCLCs. (C) Counting and statistics of embryoid bodies (*n* = 3, Lowercase letters a, b, c indicate significant statistical differences between groups, analyzed by one‐way ANOVA with Tukey's multiple comparison test *P* < 0.05). (D) Quantitative detection of the expression of marker genes in PGCLCs (*n* = 3, Lowercase letters a, b, c indicate significant statistical differences between groups, analyzed by one‐way ANOVA with Tukey's multiple comparison test *P* < 0.05). (E) Flow cytometric analysis of the positive rate of PGCLC formation. (F) Statistics of CVH positive cell rate, Data are presented as mean ± SD (*n* = 3, Lowercase letters a, b, c indicate significant statistical differences between groups, analyzed by one‐way ANOVA with Tukey's multiple comparison test *P* < 0.05).

To optimize the induction system, based on the BMP4/BMP8b/EGF induction system previously established in our laboratory and combined with the latest research on the two‐step method, we screened SCF and LIF that can enhance the induction efficiency. According to different combinations of factors, we constructed four induction systems (Fig. 3A), and the formulations are shown in Table 3. Subsequently, they will be referred to as Induction 1 to Induction 4. The induced EpiLCs were transferred to four different PGCLCs induction media. The media were replanted every 2 days, and photographs were taken to record the morphological changes of the cells and the number of EBs during the PGCLCs induction process. Morphological observations showed that during the induction process, the cells underwent proliferation, there was an increase in the number of EBs, and there was also partial formation of PGCLCs. Subsequently, the number of cells decreased, and the number of EBs also declined (Fig. 3B). Among the four systems, induction 2, 4, 3, and 1, the number of EBs is from highest to lowest (Fig. 3C). In summary, all four systems were able to induce the differentiation of EpiLCs into PGCLCs, with the number of EBs first increasing and then decreasing, reaching a peak on the 6th day. It is speculated that the PGCLCs formed using the induction 2 system after 6 days of culture have the highest efficiency.

To further investigate the optimal time for inducing EpiLCs to differentiate into PGCLCs *in vitro*, we monitored the expression of PGCLCs marker genes *Cvh*, *Kit*, and *Dazl* during the induction process. The qRT‐PCR results revealed that the expression levels of all three genes underwent a trend of initial increase and subsequent decrease, with the highest expression level observed on the 6th day. Specifically, the expression level of induction 2 was significantly higher than that of the other groups (Fig. 3D), further validating that PGCLCs induced on the 6th day achieved the best effect. Furthermore, we collected cells from the four induction systems on the 6th day, incubated them with CVH antibodies, and employed flow cytometry analysis to detect the positive cells for PGCLCs. The aim was to identify the system with the highest induction efficiency. Flow cytometry results showed that the positive cell rates for PGCLCs in each induction group were: 28.1% for induction 1, 43.7% for induction 2, 38% for induction 3, and 40.9% for induction 4 (Fig. 3E). Statistical analysis using the Tukey multiple comparison test indicated that the positive rates of PGCLCs, from highest to lowest, were in Induction Group 2, Induction Group 4, Induction Group 3, and Induction Group 1 (Fig. 3F). The specific results of pairwise comparisons are as follows: There was an extremely significant difference between Induction 1 and Induction 2 (*P* = 0.0002, *P* < 0.001); there was a significant difference between Induction 1 and Induction 3 (*P* = 0.0010, *P* < 0.05); there was an extremely significant difference between Induction 1 and Induction 4 (*P* = 0.0004, *P* < 0.01); Induction 2 and Induction 3 were nearly significant (*P* = 0.0166, *P* < 0.05); there was no significant difference between Induction 2 and Induction 4 (*P* = 0.2582); and there was no significant difference between Induction 3 and Induction 4 (*P* = 0.0868). These results corroborate the previous findings, demonstrating that the induction 2 culture system exhibits a higher induction efficiency and can be used as the subsequent culture system for inducing chicken EpiLCs to differentiate into PGCLCs *in vitro*.

### Indirect immunofluorescence staining for PGCLCs

**Fig. 4:**
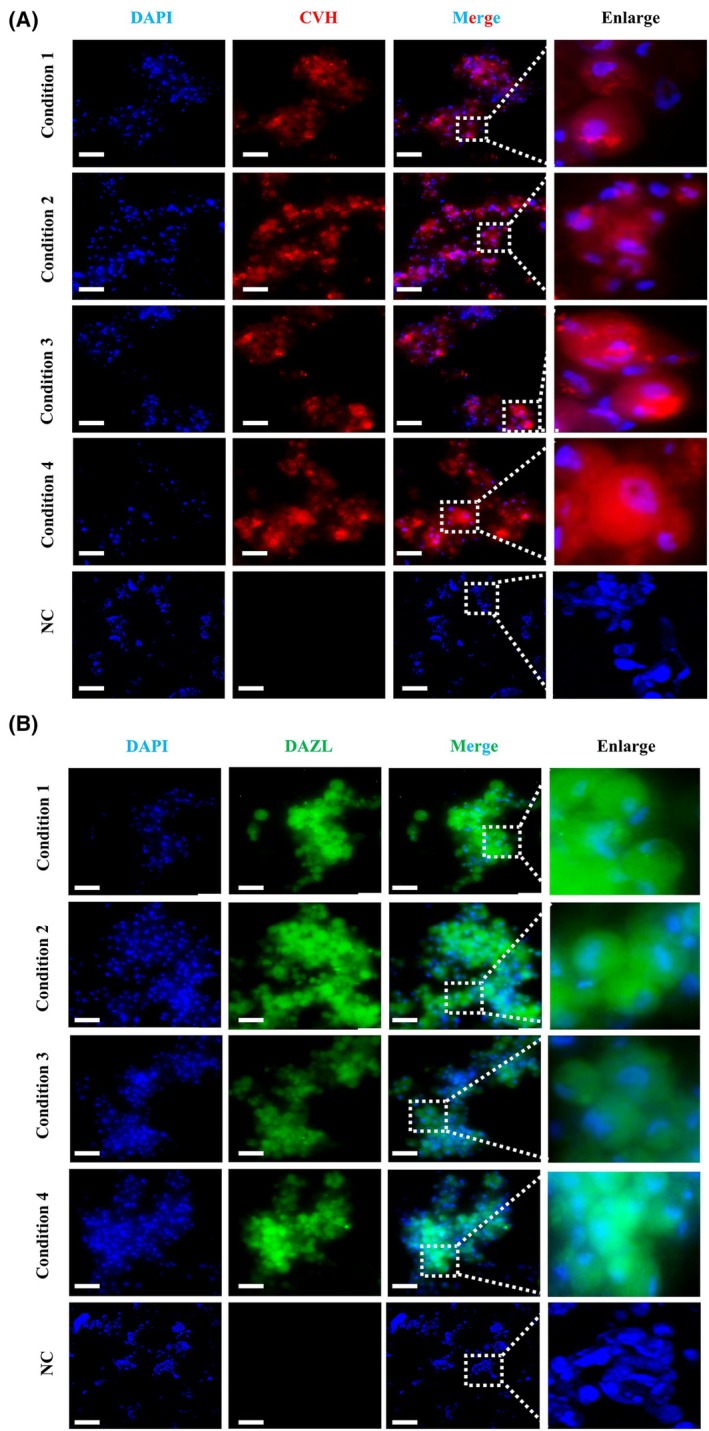
Indirect immunofluorescence staining for PGCLCs: (A) Immunofluorescence staining result of CVH on PGCLCs and negative control EpiLCs (Enlarge presents magnified views of dashed‐boxed areas, scale bar = 20 μm) (B) Immunofluorescence staining result of DAZL on PGCLCs and negative control EpiLCs (Enlarge presents magnified views of dashed‐boxed areas, scale bar = 20 μm).

To detect whether the PGCLCs formed by four *in vitro* induction systems possess PGC characteristics, the cells induced on the 6th day were subjected to fluorescent staining to detect the expression of PGC‐specific antibodies CVH and DAZL. Observation under a fluorescence microscope revealed that all four induced cell types showed ideal staining results. In terms of staining specificity, the PGC‐specific antibody CVH precisely bound to the target protein, with the cytoplasm showing an obvious red color, while no specific fluorescence staining was observed in the negative control EpiLC cells (Fig. 4A). The DAZL antibody also accurately bound to its corresponding target protein, with the cytoplasm showing a clear green color, while no specific fluorescence staining was observed in the negative control EpiLC cells (Fig. 4B). Throughout the process, there was no mislabeling of other irrelevant cells or cellular components, which strongly demonstrated the high specificity of the staining and its ability to accurately mark cells with PGC characteristics. Regarding the clarity of the staining signal, the red fluorescence signal generated by CVH labeling and the green fluorescence signal generated by DAZL labeling were extremely obvious, forming a strong and distinct contrast with the blue color of the nucleus stained by DAPI. This clear signal manifestation enabled us to easily observe and distinguish different components within the cell, fully indicating that the staining signal was strong and clear enough to truthfully and accurately reflect the expression status of relevant marker proteins in the cells. Considering the overall staining effect, common staining quality problems such as uneven staining, local staining deficiency, or over‐staining did not occur in the entire sample. After staining, the original morphology and structure of the cells remained intact and clearly distinguishable. Taken together, these satisfactory staining results indicate that the PGCLCs induced under the four systems possess similar characteristics of expressing the germ cell‐specific genes CVH and DAZL as PGCs.

### Culture and identification of PGCs and PGCLCs

**Fig. 5:**
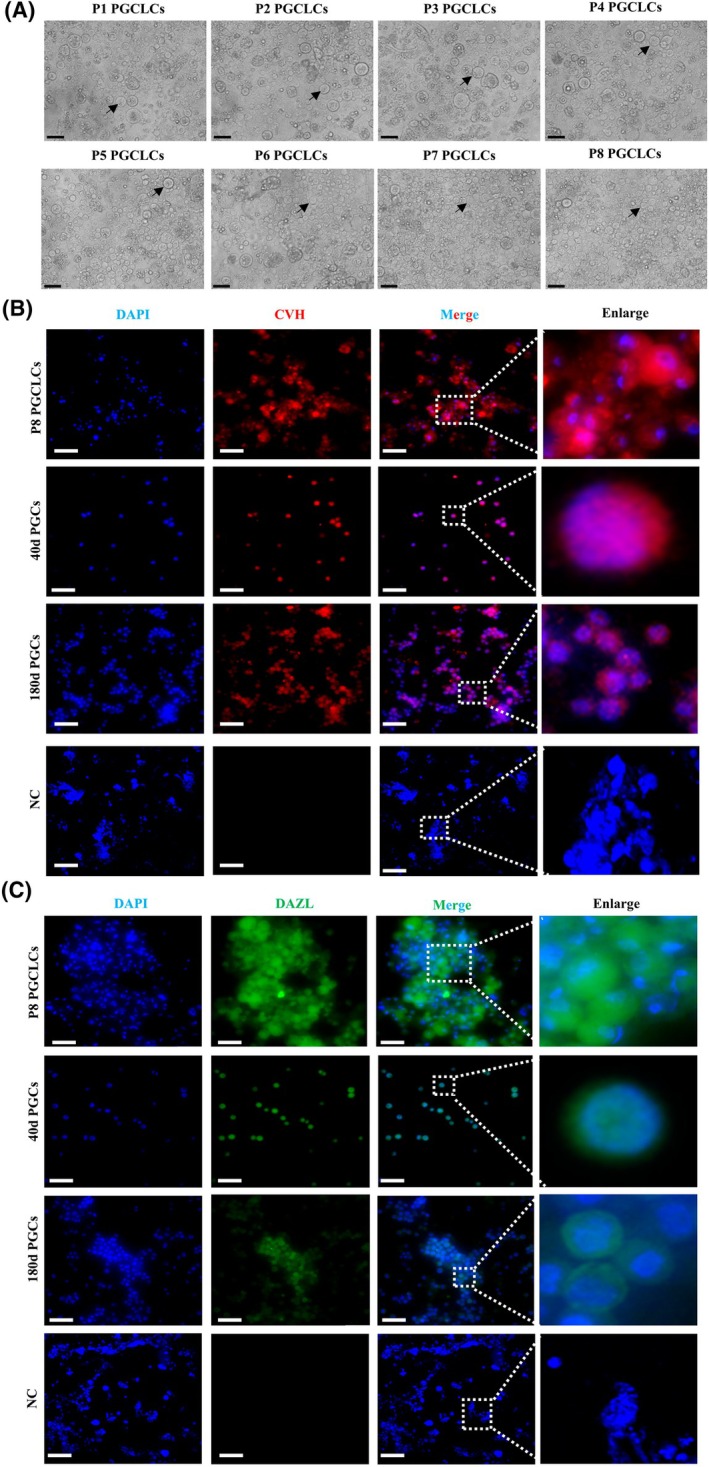
Culture and identification of PGCs and PGCLCs: (A) Morphological observation of chicken PGCLCs during long‐term culture. (B) Immunofluorescence staining result of CVH in cultured cells and negative control Epiblast cells (Enlarge presents magnified views of dashed‐boxed areas, scale bar = 20 μm). (C) Immunofluorescence staining result of DAZL in cultured cells and negative control Epiblast cells (Enlarge presents magnified views of dashed‐boxed areas, scale bar = 20 μm).

The utilization of the FACS system [15] for culturing and inducing PGCLCs demonstrated notable cell proliferation while preserving their characteristic morphology. During a culture span of up to 8 passages (equivalent to approximately 25 days), they still retained a high proliferation rate and typical cell morphology (Fig. 5A). To examine whether the PGCs and PGCLCs formed under the FACS culture system maintained their germline cell characteristics, immunofluorescence staining was performed on *in vitro* cultured F8 passage PGCLCs, 40‐day‐old, and 180‐day‐old PGCs to detect the expression of the PGCs specific antibody CVH and the specific cell surface antigen SSEA‐1. The immunofluorescence staining results showed that all three cell types could be successfully labeled by CVH and SSEA‐1. The cytoplasm appeared red due to CVH labeling (Fig. 5B), and the cell membrane appeared green due to SSEA‐1 labeling (Fig. 5C). This indicates that at the protein expression level, the induced PGCLCs are similar to the natural PGCs, suggesting that there may be commonalities in cell functions and characteristics, and providing evidence for similar gene expression indirectly. Meanwhile, the cell nuclei were stained blue by DAPI. DAPI staining revealed that the nuclei of the induced PGCLCs and the isolated PGCs were similar in morphology and distribution. Since the nucleus is the core site for storing genetic material and regulating gene expression, this similarity reflects the resemblance between the two in terms of genetic information storage and transmission, suggesting that their genomic organization and the like are similar, which is crucial for gene expression regulation and the maintenance of cell totipotency. By integrating the similarity in protein expression demonstrated by immunofluorescence staining and the similarity in nuclear genetic characteristics reflected by DAPI staining, from the perspective of cell molecular biology, as proteins are the final products of gene expression, the two aspects corroborate each other. It is reasonable to infer that the induced PGCLCs and the isolated PGCs possess similar totipotency and germline specific gene expression. This further proves that the established induction system is effective in generating PGCLCs with similar characteristics.

### Detection of *in vivo* migration ability of PGCLCs

**Fig. 6:**
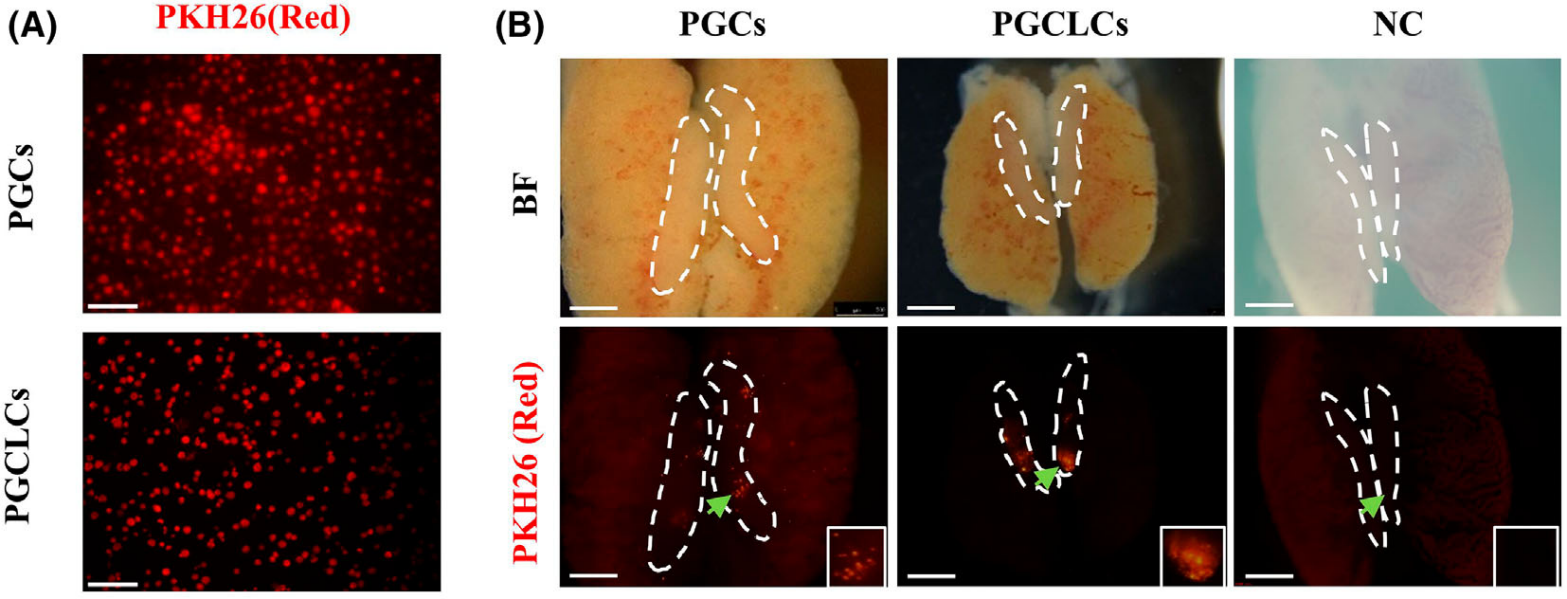
Detection of *in vivo* migration ability of PGCLCs: (A) Membrane marker results of PGCLCs and PGCs (scale bar = 50 μm). (B) Observation results of migratory fluorescence in PGCLCs and PGCs, and negative control (scale bar = 50 μm).

To detect whether the induced PGCLCs can maintain the ability to migrate to the gonad like PGCs after transplantation *in vivo*, the P8 passage PGCLCs and PGCs were labeled with PKH26 (red) fluorescent markers. The results showed that both cell types could be labeled by the reagents and remained in suspension after staining (Fig. 6A). Subsequently, the labeled cells were injected into the blood vessels of fertilized eggs after 2.5 days of incubation through the chicken embryo vasculature. The gonads were removed and separated after 7–7.5 days of incubation, and the fluorescence was observed under a fluorescence stereomicroscope. Meanwhile, negative controls were set up to exclude non‐specific interference. The results showed that both the P8 passage PGCLCs and PGCs cultured *in vitro* were able to migrate to the gonad of the recipient chicken embryos and were detected with fluorescently labeled cells (Fig. 6B). Statistical results indicated that the migration rate of PGCs was 56.5% and that of PGCLCs was 42.9% (Survival rate = Survival Count/Transplant Count, Migration Rate = Fluorescent Count/Survival Count, Table 4), suggesting that the cells cultured *in vitro* still maintained germline cell characteristics and could migrate to the recipient gonad and colonize.

**Table 4:** Statistics of cell transplantation *in vivo*.

Category	Transplant count	Survival count	Fluorescent count	Survival rate	Migration rate
PGCs	30	23	13	76.7%	56.5%
PGCLCs	30	21	9	70%	42.9%

## Discussion

Embryonic stem cells can be induced to differentiate into all cell types in the body under different conditions. The stability of embryonic stem cell lines cultured *in vitro* determines the quality and quantity of cell differentiation. Currently, the method of establishing an *in vitro* culture system for cESCs is still in the research stage Aubel [18] used STO as feeder cells and ESA as a complete medium to culture cESCs and successfully generate chimeric animals. However, due to the complexity of feeder cell production and the low purity of the resulting cells, it is difficult to culture the stem cells sustainably. Therefore, in this experiment, the culture medium was optimized by adding 2i (MEK inhibitor PD0325901 and TGFβ inhibitor SB431542) to the original factors, which promotes the proliferation of cESCs and maintains their undifferentiated state over a long period. Among them, MEK phosphorylates ERK, and the phosphorylated ERK is transferred to the nucleus to initiate the expression of pluripotent genes such as Nanog and Oct4 [19, 20]; TGFβ plays a fundamental role in evolution in various important biological processes including morphogenesis, cell fate determination, proliferation, differentiation, and apoptosis [21] After optimizing the system, the cloned shape of cESCs grown in colonies was more typical, with a more significant number and more accessible aggregation into colonies. Although these cultured cells are not strictly embryonic stem cells in the conventional sense, they still possess embryonic stem cell characteristics, maintaining an undifferentiated state and exhibiting developmental totipotency. Therefore, this study established and optimized a feeder‐free culture system for cESCs.

Compared with the research on human and mouse embryonic stem cells, there are many differences in the use of culture media. Human embryonic stem cells are often cultured in complex media containing a variety of growth factors, such as the addition of LIF, bFGF, etc., to maintain their pluripotency [22, 23]. Mouse embryonic stem cells are usually cultured in media containing serum substitutes and specific small ‐ molecule compounds. For example, in the classic 2i culture system, PD0325901 and CHIR99021 are added to inhibit related signaling pathways and stabilize the pluripotency of cells [24, 25, 26]. In this study, for cESCs, a unique medium optimization scheme was formed by adding PD0325901 and SB431542 in 2i, which effectively promoted the proliferation of cESCs and the maintenance of their undifferentiated state. In terms of cell differentiation induction, the differentiation of human embryonic stem cells into germ cells requires a specific combination of cytokines and a precise culture microenvironment [27, 28]. During the differentiation of mouse embryonic stem cells into germ cells, the demands for cytokines and signaling pathways at different developmental stages also have their own characteristics [29, 30]. In the process of cESCs differentiating into EpiLCs and PGCLCs in this study, a strategy of adding different cytokines in stages was adopted according to the characteristics of chicken cells.

Current methods for the study of ESCs differentiation into germ cells include adding cytokines, coculturing with germ cells, chemical reagent induction, transgenic induction, etc. Among these methods, cytokines can precisely regulate the cell proliferation and differentiation processes, thus improving differentiation efficiency and reducing side effects. Therefore, this study adopts a two‐step method by adding essential cytokines to the culture medium to achieve the goal. The first step is to induce cESCs to differentiate into EpiLCs. By adding bFGF, activin A, and 1% serum replacement (KSR) to the KO primary culture medium, the optimized cESCs are induced to differentiate into EpiLCs *in vitro*. bFGF is a heparin binding protein that mainly binds to FGFR on the cell membrane, promoting cell division and inducing the proliferation and differentiation of various cells [31]. Activin A maintains the undifferentiated state of cESCs by activating the expression of Nanog [32]. KSR is a synthetic substance, and studies have shown that adding KSR to the culture medium can maintain the karyotype integrity and pluripotency of mouse embryonic stem cells (mESCs) [33, 34, 35]. The second step is to induce further EpiLCs to differentiate into PGCLCs by adding different combinations of BMP4, BMP8b, EGF, LIF, and SCF to the culture medium. BMP4 supports the self‐renewal of cESCs and promotes the differentiation of cESCs into PGCs [36, 37]. EGF is a multifunctional growth factor with a robust mitogenic effect, acting on various tissue cells *in vivo* and *in vitro*. In addition, EGF can also promote the expression of some genes related to proliferation, such as myc, fos, etc., and increase the activity of DNA topoisomerase in cells. Stem cell factor, also known as c‐kit ligand, can prolong the survival time of basal stem cells, enhance their sensitivity to other cytokines, and promote the division and proliferation of stem cells. In addition, SCF can also activate telomerase activity, which is crucial for maintaining the survival of ESCs [38]. In terms of the application of cells in poultry production, cESCs and their differentiated EpiLCs and PGCLCs hold broad prospects. Combining gene ‐ editing techniques with cESCs culture allows for precise modification of poultry genes, thereby cultivating poultry breeds with excellent traits, such as enhancing the disease resistance, growth rate, and meat quality of poultry. For instance, by knocking out or editing specific genes, it is expected to breed new strains that are resistant to certain common poultry diseases, reducing disease losses during the breeding process and improving production efficiency [39, 40].

Compared with natural PGCs, the research and application of PGCLCs have opened up a new and important pathway for the conservation and innovation of germplasm resources of endangered poultry species. Natural PGCs have demonstrated significant value in the genetic breeding and germplasm conservation of poultry. However, for endangered poultry species, due to their scarce population and special reproductive cycle, obtaining natural PGCs poses great challenges. The germ cells of these rare poultry species only appear at specific embryonic development stages, and there are significant differences in the developmental processes among individuals. This leads to the fact that cell collection not only requires complex operation techniques but is also limited by the scarcity of sample resources, making it difficult to meet the demand for the quantity of cells in research and application [41]. In contrast, the directional induction technology of ESCs into PGCLCs can effectively make up for the limitations of obtaining natural PGCs. By optimizing the *in vitro* induction system, we can break through the limitations of factors such as seasons and individual development stages and stably obtain a large number of PGCLCs [42]. This technology not only provides a stable and reliable cell source for establishing cell resource banks of endangered poultry species but also creates conditions for in‐depth research on the developmental mechanisms of germ cells of endangered poultry species. Meanwhile, with the support of the optimized culture system, PGCLCs can maintain relatively stable cell characteristics and functions *in vitro*, providing a solid foundation for subsequent genetic manipulation and application [43]. The research results have notable scientific significance and application value for the long‐term conservation and sustainable utilization of germplasm resources of endangered poultry species.

In mammalian studies, ESCs‐derived PGCLCs have been confirmed to differentiate into functional germ cells and contribute to offspring production. For example, Geijsen [44] successfully induced mESCs to differentiate into PGCs, which further developed into sperm‐like cells and produced healthy offspring after transplantation; Zhou [45] induced ESCs‐derived GCLCs with meiotic competence in rats, demonstrating their *in vitro* reproductive potential. However, research in birds remains in its infancy, and whether ESCs‐induced PGCLCs can differentiate into spermatogonia/oogonia has not been reported. In this study, cPGCLCs exhibited classic PGCs markers (CVH, DAZL) and the ability to migrate and colonize gonadal ridges. However, as precursors of spermatogonia/oogonia, their potential for further differentiation requires verification. When using mammalian differentiation protocols to induce PGCLCs toward germ cells, we observed low efficiency in both *in vitro* differentiation and *in vivo* gonadal colonization. Moving forward, we will focus on exploring avian‐specific differentiation induction strategies to provide critical data for improving *in vitro* reconstruction of avian germ cells. Toyooka [46] successfully induced mESCs to differentiate into germ cells under specific culture conditions and factor induction *in vitro*. These germ cells derived from ESCs have the potential to develop into sperm or egg cells. On this basis, this study employed PKH26 red cell membrane surface markers to label P8 PGCLCs and PGCs cultured *in vitro*. Fluorescence was observed in the gonads of the recipient chicken embryos after transplantation. The results demonstrated that the P8 PGCLCs and PGCs cultured *in vitro* still maintained their germ cell characteristics and remained capable of migrating to the recipient gonad after injection into the blood vessels of the recipient chicken embryo. In the cell labeling process, we initially selected PKH26 for staining. The core purpose was to label PGCs and PGCLCs, thereby tracking their trajectories after embryonic development. Admittedly, we are aware that there is a possibility that PKH26 may stain dead cells, leading to false positive results. However, in the actual operation, we carried out the injection immediately after completing the staining process. Since dead cells do not have the ability to migrate and cannot enter the gonads to participate in the subsequent embryonic development process at all. Therefore, based on our existing experimental procedures, the probability of dead cell staining interfering with the tracking trajectories of PGCs and PGCLCs is extremely low and will not have a crucial impact on the accuracy and reliability of the experimental results. The development of this technology can provide a pathway for poultry production that does not require the isolation of PGCs. Currently, besides chickens, there is no stable PGCs culture system for other poultry species. However, the culture and induction of ESCs have the potential to effectively address issues related to performing such operations on other species in the future.

## Conclusions

In summary, this study first optimized the feeder‐free culture system for cESCs and obtained clones with AKP and SSEA‐1 activities, as well as high expression of pluripotent genes *NANOG, SOX2*, and *OCT4*. Based on this, a protocol for inducing cESCs to differentiate into PGCLCs *in vitro* was successfully established, and the system with the highest induction efficiency that was screened out is induction 2. The induced PGCLCs were able to express germ‐specific genes and could migrate and colonize in the recipient gonad. The establishment of this system will lay a solid foundation for future research on genetic improvement, disease model construction, and other studies using cESCs and PGCLCs. Additionally, this study will provide references and insights for the differentiation of cESCs into PGCLCs in other animals.

## Conflict of interest

All authors declare that there are no actual or potential conflicts of interest within 3 years prior to the submission of this work; specifically, there are no financial, personal, or other relationships with other individuals or organizations that could improperly influence, or be perceived as potentially influencing, the research work presented herein, and it is hereby stated that there are no conflicts of interest in this study.

## Author contributions

Conceptualization, BL, QZ, and JS; methodology, YN, KJ, HS, and QZ; validation, XX, KJ, and ZL; formal analysis, XX and ZL; investigation, XX, JW, GL, XL, XZ, YH, and ZL; resources, BL; data curation, XX and ZL; writing—original draft preparation, ZL; writing—review and editing, ZL, KJ, HS, and BL; supervision, BL; project administration, BL; funding acquisition, BL; all authors have read and agreed to the published version of the manuscript.

## Supporting information


**Fig. S1.** Standard curves of the expression levels of pluripotency genes.
**Fig. S2.** Agarose gel electrophoresis images after the quantitative detection of *Nanog*, *Sox2*, and *Oct4* genes in DF1 cells and ESCs.
**Fig. S3.** Standard curves for the expression levels of genes related to EpiLCs.
**Fig. S4.** Agarose gel electrophoresis image after the quantitative detection of related genes in ESCs, EpiLCs on the 1st, 2nd and 3rd days of induction, as well as Epiblast cells.
**Fig. S5.** Standard curves of the expression levels of marker genes in PGCLCs, taking the PGCLCs formed by Induction 2 as an example.
**Fig. S6.** Agarose gel electrophoresis image of the quantitative detection of marker genes in PGCLCs of cells on the 0th, 2nd, 4th, 6th and 8th days of induction, taking Induction 2 as an example.

## Data Availability

The data that support the findings of this study are available within the article and its figures, tables, and figure legends. The primer sequences used in this study are listed in Tables 1 and 2. All other relevant data, including raw qRT‐PCR results, original images of immunofluorescence staining, and flow cytometry data, are available from the corresponding author (Kai Jin, 007838@yzu.edu.cn) upon reasonable reques.
