# Supplementary material for: Establishment and optimization of the two‐step induction system for generating primordial germ cell‐like cells from chicken embryonic stem cells
Source: FEBS Open Bio. 2025 Sep 10;16(1):161–77. doi: 10.1002/2211-5463.70116 (PMC12767776; doi:10.1002/2211-5463.70116)
Supplement: Supplementary file 1 — Fig. S1. Standard curves of the expression levels of pluripotency genes. Fig. S2. Agarose gel electrophoresis images after the quantitative detection of Nanog, Sox2, and Oct4 genes in DF1 cells and ESCs. Fig. S3. Standard curves for the expression levels of genes related to EpiLCs. Fig. S4. Agarose gel electrophoresis image after the quantitative detection of related genes in ESCs, EpiLCs on the 1st, 2nd and 3rd days of induction, as well as Epiblast cells. Fig. S5. Standard curves of the expression levels of marker genes in PGCLCs, taking the PGCLCs formed by Induction 2 as an example. Fig. S6. Agarose gel electrophoresis image of the quantitative detection of marker genes in PGCLCs of cells on the 0th, 2nd, 4th, 6th and 8th days of induction, taking Induction 2 as an example. [file FEB4-16-161-s001.docx]

Supplementary Material


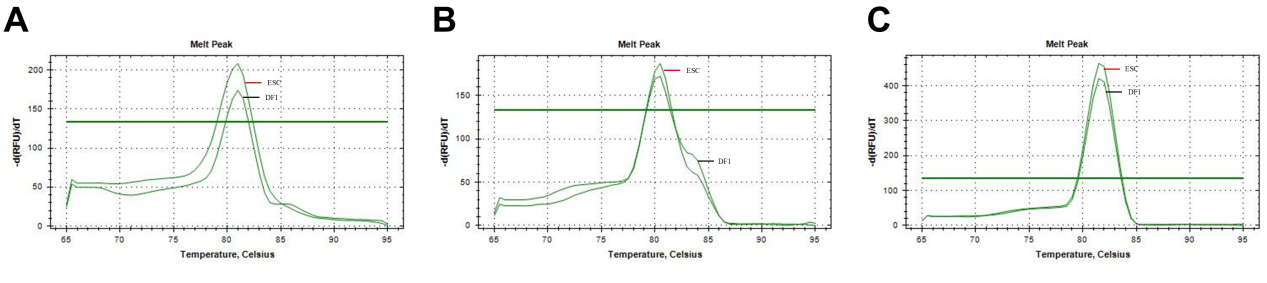


S1. Standard curves of the expression levels of pluripotency genes. (A) Standard curve of the expression level of the *Nanog* gene. (B) Standard curve of the expression level of the *Sox2* gene. (C) Standard curve of the expression level of the *Oct4* gene.


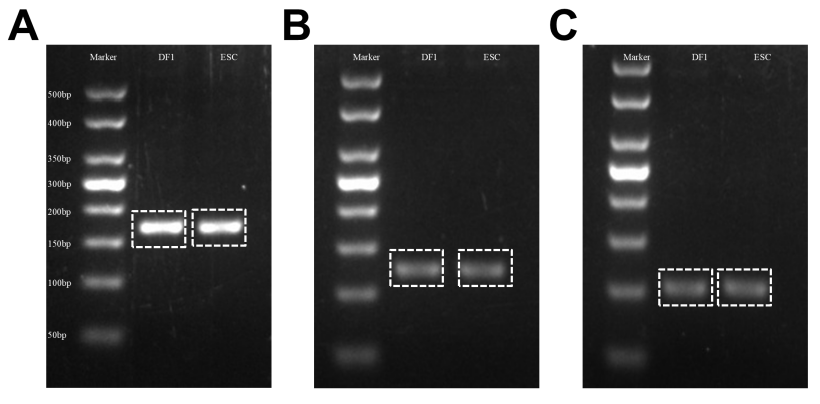


S2.Agarose gel electrophoresis images after the quantitative detection of *Nanog*, *Sox2*, and *Oct4* genes in DF1 cells and ESCs. (A)Agarose gel electrophoresis images after the quantitative detection of *Nanog* genes in DF1 cells and ESCs.(B) Agarose gel electrophoresis images after the quantitative detection of *Sox2* genes in DF1 cells and ESCs.(C)Agarose gel electrophoresis images after the quantitative detection of *Oct4* genes in DF1 cells and ESCs


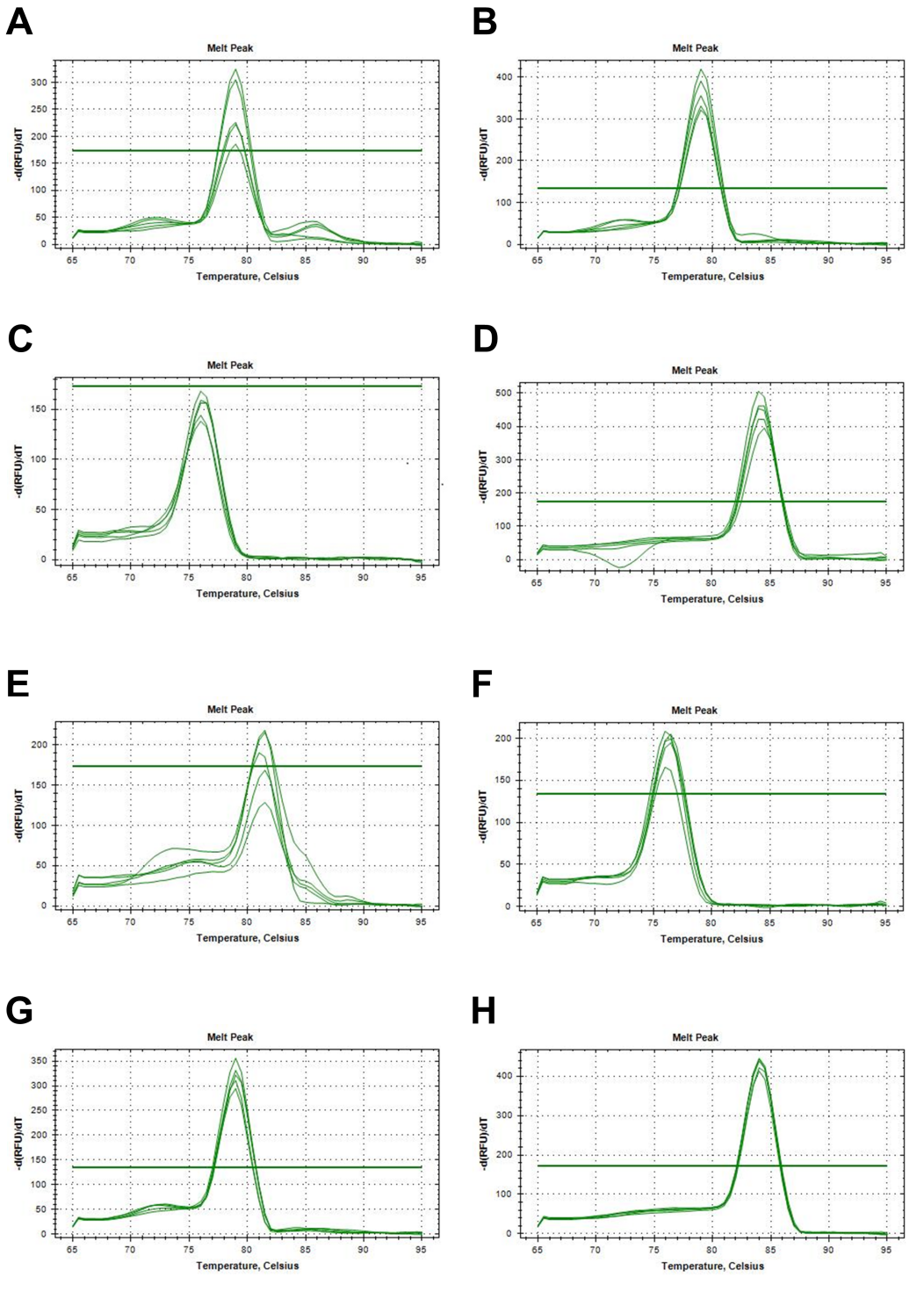


S3. Standard curves for the expression levels of genes related to EpiLCs.(A) Standard curve of the expression level of the *Pax6* gene.(B) Standard curve of the expression level of the *Eomes* gene.(C) Standard curve of the expression level of the *Vimentin* gene.(D) Standard curve of the expression level of the *Nanog* gene.(E) Standard curve of the expression level of the *Cvh* gene.(F) Standard curve of the expression level of the *C-kit* gene.(G) Standard curve of the expression level of the *Dazl* gene.(H) Standard curve of the expression level of the *Oct4* gene.


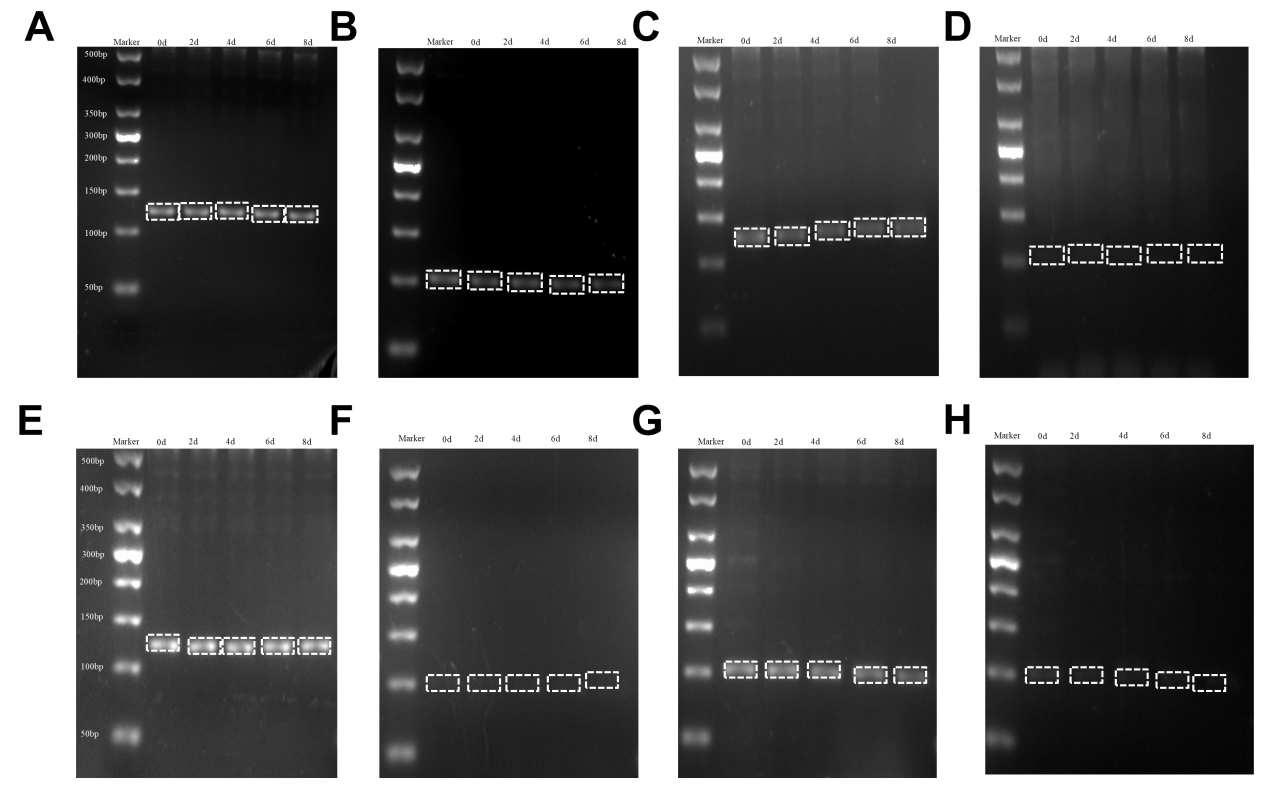


S4. Agarose gel electrophoresis image after the quantitative detection of related genes in ESCs, EpiLCs on the 1st, 2nd and 3rd days of induction, as well as Epiblast cells.(A)Agarose gel electrophoresis image after the quantitative detection of *Pax6* in ESCs, EpiLCs on the 1st, 2nd, and 3rd days of induction, as well as epiblast cells.(B)Agarose gel electrophoresis image after the quantitative detection of *Eomes* in ESCs, EpiLCs on the 1st, 2nd, and 3rd days of induction, as well as epiblast cells.(C)Agarose gel electrophoresis image after the quantitative detection of *Vimentin* in ESCs, EpiLCs on the 1st, 2nd, and 3rd days of induction, as well as epiblast cells.(D)Agarose gel electrophoresis image after the quantitative detection of *Nanog* in ESCs, EpiLCs on the 1st, 2nd, and 3rd days of induction, as well as epiblast cells.(E)Agarose gel electrophoresis image after the quantitative detection of *Cvh* in ESCs, EpiLCs on the 1st, 2nd, and 3rd days of induction, as well as epiblast cells.(F)Agarose gel electrophoresis image after the quantitative detection of *C-kit* in ESCs, EpiLCs on the 1st, 2nd, and 3rd days of induction, as well as epiblast cells.(G)Agarose gel electrophoresis image after the quantitative detection of *Dazl* in ESCs, EpiLCs on the 1st, 2nd, and 3rd days of induction, as well as epiblast cells.(H)Agarose gel electrophoresis image after the quantitative detection of *Oct4* in ESCs, EpiLCs on the 1st, 2nd, and 3rd days of induction, as well as epiblast cells.


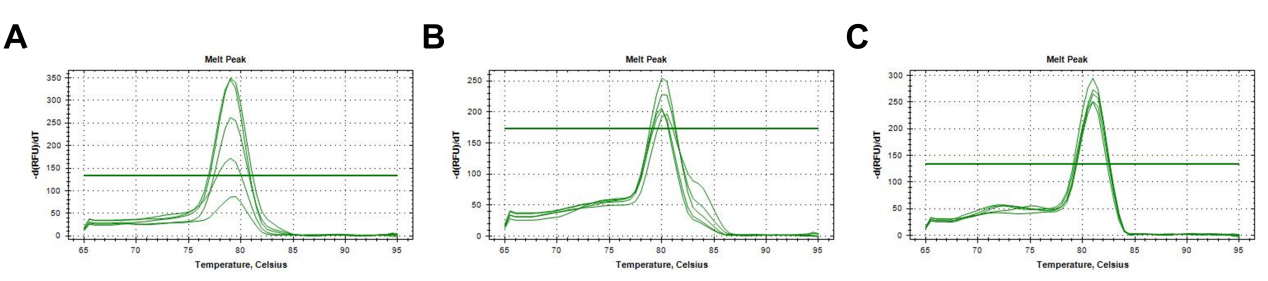


S5. Standard curves of the expression levels of marker genes in PGCLCs, taking the PGCLCs formed by Induction 2 as an example.(A) Standard curve of the expression level of the *Dazl* gene.(B) Standard curve of the expression level of the *C-ki*t gene.(C) Standard curve of the expression level of the *Cvh* gene.


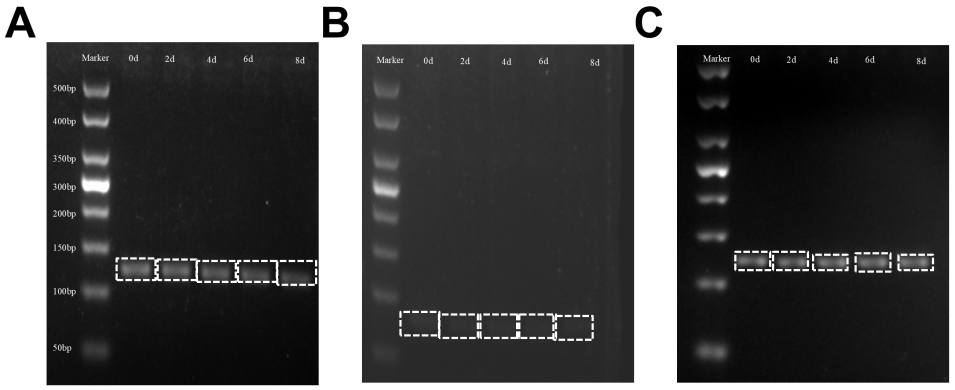


S6. Agarose gel electrophoresis image of the quantitative detection of marker genes in PGCLCs of cells on the 0th, 2nd, 4th, 6th and 8th days of induction, taking Induction 2 as an example. (A) Agarose gel electrophoresis image after the quantitative detection of *Dazl* gene in cells on the 0th, 2nd, 4th, 6th, and 8th days of induction.(B)Agarose gel electrophoresis image after the quantitative detection of *C-kit* gene in cells on the 0th, 2nd, 4th, 6th, and 8th days of induction.(C)Agarose gel electrophoresis image after the quantitative detection of *Cvh* gene in cells on the 0th, 2nd, 4th, 6th, and 8th days of induction
